# Efficacy and safety of a single intra-articular injection of 6 ml Hylan G-F 20 compared to placebo in Chinese patients with symptomatic knee osteoarthritis: C-SOUND study, a 26-week multicenter double-blind randomized placebo-controlled trial in China

**DOI:** 10.1186/s12891-021-04252-2

**Published:** 2021-05-08

**Authors:** Yan Ke, Wenxue Jiang, Yongsheng Xu, Yajun Chen, Qingsong Zhang, Qingyun Xue, Jianhao Lin, Wilson Ngai, Gaowei Nian, Mir Sohail Fazeli, Yao Xie, Zhenan Zhu

**Affiliations:** 1 Peking University People’s Hospital, Beijing, China; 2 Tianjin First Central Hospital, Tianjin, China; 3 Inner Mongolia People’s Hospital, Hohhot City, China; 4 Tianjin Medical University General Hospital, Tianjin, China; 5 Wuhan Fourth People’s Hospital; Puai Hospital, Tongji Medical College, Huazhong University of Science and Technology, Wuhan, China; 6 Beijing Hospital, Beijing, China; 7 Sanofi US, Bridgewater, NJ, USA; 8 Sanofi China, Shanghai, China; 9 Evidinno Outcomes Research Inc., Vancouver, British Columbia Canada; 10 Shanghai Ninth People’s Hospital, Shanghai JiaoTong University School of Medicine, No.639 Zizaoju Road, Huangpu District, Shanghai, China

**Keywords:** Knee osteoarthritis, Single 6 ml Hylan G-F 20 injection, Clinical outcomes, Placebo effect

## Abstract

**Background:**

Single 6 ml Hylan G-F 20 injection, is indicated for knee osteoarthritis patients who have failed to respond to non-pharmacologic therapy and/or simple analgesics. To obtain more thorough understanding of the clinical efficacy and safety, a randomized clinical trial was conducted comparing intra-articular (IA) administration of single 6 ml Hylan G-F 20 injection versus placebo in knee OA patients of Chinese ethnicity.

**Methods:**

This was a randomized, multi-center, double-blind, placebo-controlled clinical trial conducted in 21 centers across China. Four hundred forty adults with knee OA received a single 6 ml Hylan G-F 20 or placebo injection and were evaluated for clinical efficacy and safety outcomes over 26 weeks. Western Ontario and McMaster Universities OA (WOMAC) A1 index, treatment-emergent adverse events (TEAEs) and standard safety parameters were measured at pre-injection, and at weeks 1, 4, 8, 12, 16, 20 and 26 post-injection.

**Results:**

Four hundred forty patients (male: 98 [22.3%]; female: 342 [77.7%]) were randomized. The mean age [standard deviation (SD)] was 61.5 (7.9) years. All patients were of East Asian ethnicity. Mean WOMAC A1 score at baseline was 5.3 (1.2) and 5.2 (1.3) in single 6 ml Hylan G-F 20 injection and placebo groups, respectively. Significant reductions of WOMAC A1 score were observed in both treatment groups when compared to baseline at 26 weeks post-injection, with the mean reduction of [standard error (SE)/percentage] -2.146 (0.108)/− 40.5% and − 2.271 (0.110) /− 43.7% in the single 6 ml Hylan G-F 20 injection and the placebo groups, respectively. Additionally, clinically important reductions in pain at 26 weeks was reported in 67.0 and 68.2% in single 6 ml Hylan G-F 20 injection and placebo groups (*p* = 0.36). Regarding safety, TEAEs were similar between the two treatment groups (hylan G-F 20 single: 61.5%; placebo: 64.5%).

**Conclusions:**

While the magnitude of the effect of a single 6 ml Hylan G-F 20 injection in this study is consistent with previously published literature with respect to the efficacy and safety of the drug, the current study shows a strong IA placebo effect and did not established superiority of single 6 ml Hylan G-F 20 injection over IA placebo in Chinese knee OA patients.

**Trial registration:**

Prospectively registered Jun 16, 2017 at www.clinicaltrials.gov (NCT03190369).

**Supplementary Information:**

The online version contains supplementary material available at 10.1186/s12891-021-04252-2.

## Introduction

Osteoarthritis (OA) is a highly prevalent degenerative disease, resulting in cartilage degradation, joint effusion, swelling, and pain within the affected joint, as well as functional limitation [1, 2]. The knee OA is often associated with moderate to severe disability and presents significant burden to the health care system [3]. Age, sex, body mass index, and injuries such as meniscus and anterior cruciate ligament tears have been reported as factors influencing the onset of knee OA [4, 5]. The purposes of knee OA treatment are to relieve pain, delay disease progression, correct deformity, improve or restore joint function and improve life quality of patients [6].

The evidence with respect to the efficacy of viscosupplementation with hyaluronic acid (HA) on OA of the knee is inconclusive with some studies suggesting it as an effective treatment for mild to moderate knee OA [7–11] while others claiming no difference between viscosupplementation with HA and intra-articular placebo in management of knee OA patients [12, 13]. HA is a viscoelastic molecule with beneficial effects within the synovial fluid, including chondroprotective properties, lubrication and shock absorption, anti-inflammatory effects, proteoglycan synthesis and scaffolding, and subchondral protection [14]*.* Hylan G-F 20 is a high molecular weight, cross-linked derivative of hyaluronan (extract of chicken comb) viscosupplementation. It is currently used in North America and Europe for the treatment of pain associated with knee OA under the trade names Synvisc® and/or Synvisc One®. Synvisc® is administered as 3 doses of 2 ml Hylan G-F 20 weekly injection (16 mg hylan polymer) while Synvisc One® is administered as a single injection of 6 ml Hylan G-F 20 (48 mg hylan polymer). Previously, SOUND trial demonstrated that a single injection of 6 ml of Hylan G-F 20 was safe and effective in providing symptomatic relief for up to 26 weeks in 253 European patients with primary knee OA. There was a statistically significant estimated treatment difference (− 0.15, *p* = 0.047) between the treatment and the placebo groups in the primary efficacy endpoint WOMAC A. This trial established a favorable risk/benefit profile of single 6 ml Hylan G-F 20 injection in patients with symptomatic primary OA of the knee [8]. Prior to the SOUND study, a multi-center, randomized, double-blind controlled clinical trial was conducted to evaluate the efficacy and safety of 3 doses of 2 ml Hylan G-F 20 weekly injection, in 110 chronic idiopathic knee OA patients. Results showed that 3 doses of 2 ml Hylan G-F 20 weekly injection significantly outperformed placebo for all outcomes measured using a visual analogue scale/score (VAS). Those outcomes included pain during weight-bearing movement, night pain, and pain during the most painful knee movement [10]. In North America, a randomized, double-blind, 26-week, multi-center study was conducted to compare one (3 weeks) or two courses (6 weeks) of 2 ml Hylan G-F 20 weekly injection with sham injections in 120 knee OA patients. This pivotal trial demonstrated the superiority of 2 ml Hylan G-F 20 weekly injection (one and two courses) over IA-placebo in pain with motion (*p* < 0.05), as well as superiority of two courses of 3 doses of 2 ml Hylan G-F 20 weekly injection over the one course of Hylan G-F 20 injection. No systemic adverse events were reported throughout the study period [15].

Although there have been many clinical trials conducted on the use of Hylan G-F 20 injection to date, none of them has been conducted in Chinese OA patients [8, 10, 15, 16]. Due to the lack of clinical trials conducted on HA in knee OA patients in China, the cultural, environmental and sociopsychological difference between patients living in China and aboard, it is important to investigate the efficacy and safety of a single 6 ml Hylan G-F 20 injection versus placebo in the Chinese population. For this purpose, we conducted a randomized multi-center, double-blind, placebo-controlled clinical trial in China (C-SOUND trial) in which a single 6 ml Hylan G-F 20 injection was compared to placebo for the treatment of knee OA over a period of 26 weeks.

## Method/design

### Patient population

Ethics committee approvals (Institutional Review Board, IRB number: 2017PHA036–01) and written informed consents from patients were obtained. Included patients were those aged 40 to 80 years, with grade I to III Kellgren-Lawrence OA of the knee, confirmed by standard X-ray up to 3 months before screening. Patients were required to meet the American College of Rheumatology (ACR) criteria for knee OA, had a Western Ontario and McMaster Universities Osteoarthritis Index (WOMAC) A1 Numerical Rating Scale (NRS) score of between 4.0 and 8.0 at baseline, and failed to respond to non-pharmacologic therapy and/or simple analgesics. The ACR criteria [17] are in line with Chinese Orthopedic Association Criteria [18].

Patients were excluded if they had moderately severe or severe depression as indicated by Patient Health Questionnaire-9 (PHQ-9) total score of ≥15 or a score of > 0 on item # 9, severe anxiety, or severe insomnia as indicated by a score from four questionnaires (pain DETECT, Patient Health Questionnaire-9, Generalized Anxiety Disorder-7, and Insomnia Severity Index) at the screening visit [19]. Patients who had prior knee surgery, or previous IA treatment with corticosteroids, local anesthetic agents or viscosupplementation agents to the target knee were excluded. Patients with scores of contralateral knee pain (if present) greater than 3.0 NRS, or those with ipsilateral hip OA, concomitant inflammatory disease, or other conditions that affected the joints were also excluded.

### Study treatment

Hylan G-F 20 is a sterile, nonpyrogenic, elastoviscous fluid containing hylan polysaccharide (single 6 ml Hylan G-F 20 injection, 6-mL, 48 mg hylan polymer, Sanofi). Hylan is a cross-linked derivative of hyaluronan, a natural polysaccharide (glycosaminoglycan) responsible for the elastoviscosity of synovial fluid. The hydration fluid is isotonic physiological sodium chloride solution. Six milliliters of hylan G-F 20 and the placebo control (PBS, phosphate buffer saline, pH 7.2) syringes were labeled in accordance with the local regulatory specifications and requirements. The syringes were packaged into kits that appeared identical.

### Study design

A randomized, multi-center, double-blinded, placebo-controlled clinical trial was conducted in 21 centers across China. Randomization occurred only after patients provided written informed consent, completed all screening visit procedures, and met the requirements of designated inclusion and exclusion criteria. The study was performed in accordance with the principles of Good Clinical Practice guidelines (ICH HARMONISED GUIDELINE INTEGRATED ADDENDUM TO ICH E6(R1): GUIDELINE FOR GOOD CLINICAL PRACTICE ICH E6(R2) ICH Consensus Guideline) [20]. The study protocol was registered into the US clinical trial registry under NCT03190369. The randomization and treatment allocation were performed centrally by an interactive voice response system (IVRS) with simple sequential allocation from blocked randomization schedule without stratifying factors (including site). The block size was 4 and the allocation ratio was 1:1. Patients and evaluators were blinded to the treatment received. The dose and regimen employed in this study, as well as that of US/EU pivotal study (SYNV00704, SOUND study), were decided based on a pilot study (SYNV00502) conducted by SANOFI [8, 16]. The technique for injection followed a standardized method of aseptic no touch technique.

The primary endpoint of this trial included change from baseline in WOMAC A1 (pain sub-scale, measured while walking) over 26 weeks for single 6 ml Hylan G-F 20 injection when compared to placebo. The sample size calculations were based on the primary efficacy variable of WOMAC A1 over 26 weeks, with the following assumptions: 0.75 mean difference in treatment effect of single 6 ml Hylan G-F 20 injection on the change from baseline in WOMAC A1 over 26 weeks, compared to placebo and a t-test at a 2-sided 5% significance level with 90% power. Secondary outcomes included change in WOMAC A1 measured as a 7-day average, WOMAC A, patient global assessment (PTGA), and clinical observer global assessment (COGA) over 26 weeks. Additionally, percentages of positive responders (clinically important reductions in pain) over 26 weeks, defined as a ≥ 2-point improvement from baseline in WOMAC A1 NRS, was measured as a secondary outcome. Generalized estimating equation (GEE) modeling was used for the analysis of WOMAC A 1 responders (≥2 point improvement on NRS Scale). Each responder (yes/no) endpoint evaluated at multiple post-baseline visits was analyzed using GEE for binary outcomes.

Safety outcomes were assessed through evaluation of incidence of treatment-emergent adverse events (TEAE) and changes in standard safety parameters over the entire study period. Upon entering the trial, patients were asked to provide informed consent, and were then asked to enter a medication washout period (those with a half-life > 5 h) of up to 14 days. Following wash-out, patients were reassessed for eligibility, and received study treatment. Patients were asked to report their daily pain using a patient diary. Follow-up visits was performed at weeks 1, 4, 8, 12, 16, 20, and 26. Changes of quality of life from baseline to weeks 4, 8, 12, 16, 20, and 26 were assessed by European Quality of Life-5 Dimensions (EQ-5D).

### Concomitant medications and treatments

On an as-needed basis in a tiered manner, the following therapies were allowed as rescue medication in case of unbearable pain (eg, worsening of OA symptoms in the target knee) during the study period: 1) Acetaminophen (500 mg, up to 3000 mg/day), 2) Acetaminophen (325 mg)/oxycodone (5 mg, up to 1 tablet 4 times daily), or 3) Acetaminophen (325 mg)/tramadol (37.5 mg, up to 1 tablet 6 times daily). However, rescue medication was not to be taken within 48 h prior to any study visit.

### Data collection and analysis

Statistical reports were generated using SAS version 9.4 (SAS Institute, Cary, NC, USA). Descriptive statistics of baseline characteristics was provided for continuous data using mean, standard deviation (SD), median, minimum, and maximum for each treatment group. Categorical and ordinal data were summarized using the number and percentage of patients in each treatment group. The efficacy analysis was performed on the modified intent-to-treat (mITT) population containing all randomized and treated patients as randomized. Two patients in the single 6 ml Hylan G-F 20 injection group were excluded from mITT because they didn’t receive any treatment. The statistical test for the primary efficacy endpoint, change from baseline in WOMAC A1 over 26 weeks for single 6 ml Hylan G-F 20 injection compared to placebo, was 2-sided at α level of 0.05, based on a repeated-measures analysis of covariance (ANCOVA) that was used to test for differences in treatment efficacy. Same test was used for the analysis of secondary effectiveness outcomes, change from baseline in 7-day average WOMAC A1, WOMAC A, PTGA, and COGA over 26 weeks. For the analysis of WOMAC A1 responders, GEE model was fitted to the responder data and included terms for baseline measure, site, visit, treatment group and a visit-by-treatment group interaction. Hypothesis testing was performed using least squares means based on the linear predictor of the model.

All safety analyses were performed on the safety population. All AEs (including serious AEs and AEs with pre-specified monitoring) were associated with primary system-organ-class (SOC) using the version of Medical Dictionary for Regulatory Activities (MedDRA version 21.1). The sample size (n) and percentage (%) of patients experiencing an AE were presented. AE incidence tables were presented by SOC and PT (preferred term), then were sorted in alphabetical order for each treatment group. Multiple occurrences of the same event in the same patient were counted only once in the tables within a treatment phase. The denominator for computation of percentages was the safety population within each treatment group; no imputation was performed for missing safety data.

## Results

### Patients characteristics

Across all sites, 524 patients were screened, of whom 84 patients (16.0%) failed the screening. The reasons for failure were inability to meet inclusion and exclusion criteria such as missing information on the WOMAC A1 NRS score, modified Kellgren-Lawrence Numerical Grading System of Grade I-III, signature of informed consent format, pain DETECT Questionnaire score > 18 and laboratory examination of hepatic function as well as unwilling to continue due to personal reasons. The remaining 440 patients were enrolled and randomized. Of 440 patients who were randomized, 2 patients (0.9%) in the single 6 ml Hylan G-F 20 injection group were not treated, due to poor compliance to protocol. Among 438 patients included in the mITT population, six patients (2.7%) from the single 6 ml Hylan G-F 20 injection group and 1 patient (0.5%) from the placebo group discontinued due to poor compliance to protocol or other reasons. Two hundred and twelve patients (96.4%) in the single 6 ml Hylan G-F 20 injection group and 219 patients (99.5%) in the placebo group completed the 26-week follow-up period.

**Fig. 1:**
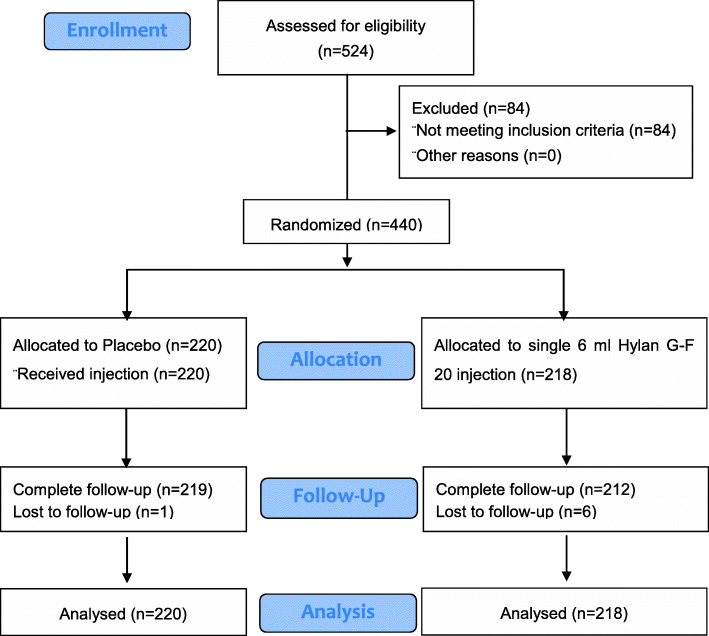
Flowchart of study population recruitment

Efficacy and safety analyses were performed on the safety population, which included 218 patients in the single 6 ml Hylan G-F 20 injection group and 220 patients in the placebo group (Fig. 1). Demographic characteristics of patients in the randomized population collected at baseline are presented in Table 1. Demographic and disease characteristics in the randomized population were generally balanced across the two treatment arms. Of the 440 randomized patients, 98 (22.3%) of them were male and 342 (77.7%) were female. The mean age of the randomized patients was 61.5 years. A total of 269 patients were > 60 years of age. All patients were of Asian ethnicity. The mean BMI was 25.48 (3.18) kg/m^2^. Medical and surgical history of patients were generally balanced between the treatment groups. Of the 440 randomized patients, 219 patients (49.8%) had OA in the left knee, while the other 221 patients (50.2%) had OA in the right knee. A total of 55 patients (12.5%) were Grade I, of which 31 patients were in the single 6 ml Hylan G-F 20 injection group and 24 patients were in the placebo group. A total of 221 patients (50.2%) had a Kellgren-Lawrence Numerical Grade of II at screening or baseline, with 105 from (in) the single 6 ml Hylan G-F 20 injection group and 116 patients from (in) the placebo group. Eight-four (38.2%) and 80 (36.4%) patients in the single 6 ml Hylan G-F 20 injection and placebo groups were Grade III respectively.

**Table 1:** Demographics and patient characteristics at screening or baseline

	Placebo (***N*** = 220)	Single 6 ml Hylan G-F 20 injection (***N*** = 220)	All (***N*** = 440)
**Gender [n (%)]**			
Male	48 (21.8)	50 (22.7)	98 (22.3)
Female	172 (78.2)	170 (77.3)	342 (77.7)
**Age (year)**			
Mean (SD)	61.6 (7.8)	61.5 (7.9)	61.5 (7.9)
Median	63.0	62.0	63.0
Min; Max	42; 79	40; 79	40; 79
**Age group [n (%)]**			
< =60	79 (35.9)	92 (41.8)	171 (38.9)
> 60	141 (64.1)	128 (58.2)	269 (61.1)
**Race [n (%)]**			
Asian	220 (100)	220 (100)	440 (100)
**Ethnicity [n (%)]**			
Not Hispanic or Latino	220 (100)	220 (100)	440 (100)
**Baseline BMI (kg/m**^**2**^**)**			
Mean (SD)	25.39 (3.29)	25.57 (3.07)	25.48 (3.18)
Median	25.39	25.23	25.28
Min; Max	16.5; 36.2	18.3; 34.4	16.5; 36.2
**Grade according to modified Kellgren-lawrence numerical [n (%)]**			
0	0	0	0
I	24 (10.9)	31 (14.1)	55 (12.5)
II	116 (52.7)	105 (47.7)	221 (50.2)
III	80 (36.4)	84 (38.2)	164 (37.3)
IV	0	0	0
**WOMAC A1 score (0–10)**			
Number	220	220	440
Mean (SD)	5.2 (1.3)	5.3 (1.2)	5.3 (1.3)

*SD* standard deviation

### Clinical outcomes

#### Change of WOMAC A1 scores from baseline over 26 weeks

**Fig. 2:**
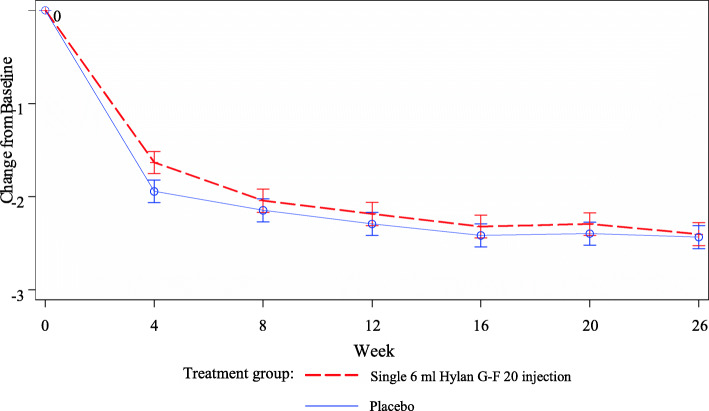
WOMAC A1: Least square Means (SE) for change from baseline over time. Least-square (LS) means and standard errors (SE) are taken from repeated measures of Covariance analysis. The model includes treatment groups (Single 6 ml Hylan G-F 20 injection and placebo), site, visit and visit by treatment interaction, as well as the baseline WOMAC A1 score as a covariate. Included are patients who have measurements at baseline and at least one post-baseline value

Baseline values with mean [standard deviation (SD)] for WOMAC A1 score were similar between single 6 ml Hylan G-F 20 injection groups [5.3 (1.2)] and placebo [5.2 (1.3)]. Both single 6 ml Hylan G-F 20 injection and placebo induced a remarkable reduction in WOMAC A1 score at all visits and across the 26 weeks. The change of least squares (LS) mean (standard error [SE]) in WOMAC A1 from baseline over 26 weeks was − 2.146 ± 0.108 and − 2.271 ± 0.110 in the 6 ml Hylan G-F 20 and placebo groups, respectively. However, there was no difference between the two groups (*p* = 0.36, Table 2). Figure 2 shows the least square mean value for WOMAC A1 for all patients who had measurements at baseline and at least one post-baseline value. Both the treatment and placebo arm demonstrated clinical meaningful improvements in the primary endpoint of WOMAC A1 scores from baseline over 26 weeks (− 40.5% in the treatment arm vs. -43.7% in the placebo arm) as the OMERACT-OARSI defined responder as 20% improvement in pain [21].

**Table 2:** Mean change in WOMAC A1 from baseline over 26 weeks

	Placebo (***N*** = 220)	Single 6 ml Hylan G-F 20 injection (***N*** = 218)
**Baseline**		
Number	220	216
Mean (SD)	5.2 (1.3)	5.3 (1.2)
Median	5.0	5.0
Min; Max	0; 8	1; 8
**Change from baseline over 26 weeks**		
LS Mean (SE)	−2.271 (0.110)	−2.146 (0.108)
LS Mean Difference (SE) vs. Placebo		0.125 (0.137)
95% CI		(−0.144 to 0.395)
*P*-value		0.3610

Least-square (LS) means, standard errors (SE) and *p*-value are taken from repeated measures of Covariance analysis. The model includes treatment groups (Single 6 ml Hylan G-F 20 injection and placebo), site, visit and visit by treatment interaction, as well as the baseline WOMAC A1 score as a covariate. Included are patients who have measurements at baseline and at least one post-baseline value

#### Other efficacy outcomes

Clinically important reductions in pain at 26 weeks were reported in 67.0% of those treated with single 6 ml Hylan G-F 20 injection and 68.2% of those treated with placebo. Also, the difference between the LS mean of the change from baseline in 7-day average WOMAC A1 in single 6 ml Hylan G-F 20 injection versus placebo groups over 26 weeks was not statistically significant (difference: 0.099, 95% CI: − 0.166 to 0.364, *p* = 0.4637, Table 3). Analyses of the changes from baseline over 26 weeks in PTGA and COGA also showed no statistical difference between the two treatment groups (Tables 4 and 5). The number of WOMAC A1 responders over 26 weeks was not different between single 6 ml Hylan G-F 20 injection-treated patients (146 patients) and the placebo (150 patients, estimate of common odds ratio: 0.83, 95% CI: 0.58 to 1.19, *p* = 0.68). This trend was consistent for all the visits. There were consistently more responders than non-responders in both treatment groups.

**Table 3:** Mean change in 7-day average WOMAC A1 from baseline over 26 weeks

	Placebo (***N*** = 220)	Single 6 ml Hylan G-F 20 injection (***N*** = 218)
**Baseline**		
Number	220	216
Mean (SD)	5.34 (1.03)	5.48 (1.07)
Median	5.14	5.36
Min; Max	3.7; 8.0	3.7; 8.3
**Change from baseline over 26 weeks**		
LS Mean (SE)	−2.275 (0.108)	−2.176 (0.106)
LS Mean Difference (SE) vs. Placebo		0.099 (0.135)
95% CI		(−0.166 to 0.364)
*P*-value		0.4637

Least-square (LS) means, standard errors (SE) and *p*-value are taken from repeated measures of Covariance analysis. The model includes treatment groups (Single 6 ml Hylan G-F 20 injection and placebo), site, visit and visit by treatment interaction, as well as the baseline 7-day average WOMAC A1 score as a covariate. Included are patients who have measurements at baseline and at least one post-baseline value

**Table 4:** Mean change in PTGA from baseline over 26 weeks

	Placebo (***N*** = 220)	Single 6 ml Hylan G-F 20 injection (***N*** = 218)
**Baseline**		
Number	220	216
Mean (SD)	5.7 (1.4)	5.8 (1.4)
Median	6.0	6.0
Min; Max	2; 10	2; 9
**Change from baseline over 26 weeks**		
LS Mean (SE)	−2.138 (0.104)	−2.144 (0.102)
LS Mean Difference (SE) vs. Placebo		−0.006 (0.130)
95% CI		(−0.262 to 0.250)
P-value		0.9621

Least-square (LS) means, standard errors (SE) and *p*-value are taken from repeated measures of Covariance analysis. The model includes treatment groups (Single 6 ml Hylan G-F 20 injection and placebo), site, visit and visit by treatment interaction, as well as the baseline PTGA score as a covariate. Included are patients who have measurements at baseline and at least one post-baseline value

**Table 5:** Mean change in COGA from baseline over 26 weeks

	Placebo (***N*** = 220)	Single 6 ml Hylan G-F 20 injection (***N*** = 218)
**Baseline**		
Number	220	216
Mean (SD)	5.5 (1.3)	5.7 (1.2)
Median	5.0	6.0
Min; Max	2; 9	2; 9
**Change from baseline over 26 weeks**		
LS Mean (SE)	−2.145 (0.095)	−2.225 (0.094)
LS Mean Difference (SE) vs. Placebo		−0.080 (0.120)
95% CI		(−0.316 to 0.155)
*P*-value		0.5036

Least-square (LS) means, standard errors (SE) and *p*-value are taken from repeated measures of Covariance analysis. The model includes treatment groups (Single 6 ml Hylan G-F 20 injection and placebo), site, visit and visit by treatment interaction, as well as the baseline COGA score as a covariate. Included are patients who have measurements at baseline and at least one post-baseline value

The amount and days of permitted pain rescue medication use at each visit were generally balanced between the two treatment groups. There was no difference in quality-of-life measures between the two treatment groups at each visit.

### Safety outcomes

All patients (218 and 220 in single 6 ml Hylan G-F 20 injection group and placebo, respectively) received the full injection volume of 6 ml. Fifty-four patients (24.8%) from the single 6 ml Hylan G-F 20 injection group and 58 patients (26.4%) from the placebo group had fluid effusion or withdrawal. Overall, frequency of TEAEs was comparable between the two treatment groups (6 ml hylan G-F 20, *n* = 134, 61.5%; placebo, *n* = 142, 64.5%). There were 16 patients (7.34%) in the single 6 ml Hylan G-F 20 injection group and 16 patients (7.27%) in the placebo group with TEAEs considered to be related to the injections. The most frequently reported TEAEs related to injection were musculoskeletal and connective tissue disorders, of which arthralgia (7 patients [3.2%] in each group) and peripheral joint swelling (8 patients [3.7%] and 2 patients [0.9%] in the single 6 ml Hylan G-F 20 injection and the placebo group respectively) were most reported (Table 6). There were no serious TEAEs, TEAEs leading to death, or TEAEs leading to treatment discontinuation over the course of the study.

**Table 6:** General disorders and administration site conditions. Number (%) of patients with TEAE(s) regardless of relationship and related to treatment by Primary SOC, and PT

Primary System Organ Class /Preferred Term n (%)	Placebo (***N*** = 220)		Single 6 ml Hylan G-F 20 injection (***N*** = 218)	
	All	Related to Placebo injections	All	Related to Hylan G-F 20 injections
Arthralgia	24 (10.9)	7 (3.2)	26 (11.9))	7 (3.2)
Asthenia	0	0	3 (1.4)	0
Injection site joint pain	1 (0.5)	1 (0.5)	2 (0.9)	1 (0.5)
Injection site pain	1 (0.5)	0	2 (0.9)	1 (0.5)
Joint swelling	4 (1.8)	2 (0.9)	16 (7.3)	8 (3.7)
Pyrexia	0	0	2 (0.9)	0
Axillary pain	0	0	1 (0.5)	0
Chest discomfort	1 (0.5)	0	1 (0.5)	0
Injection site edema	0	0	1 (0.5)	1 (0.5)
Injection site swelling	0	0	1 (0.5)	1 (0.5)
Non-cardiac chest pain	0	0	1 (0.5)	0
Edema peripheral	0	0	1 (0.5)	1 (0.5)
Chills	1 (0.5)	0	0	0
Injection site joint swelling	1 (0.5)	1 (0.5)	0	0
Malaise	1 (0.5)	0	0	0
Puncture site hemorrhage	1 (0.5)	0	0	0
Thirst	1 (0.5)	0	0	0

*TEAE* Treatment emergent adverse event, *SOC* System organ class, *PT* Preferred term MedDRA 21.1 n (%) = number and percentage of patients with at least one TEAE. Table sorted by SOC internationally agreed order and by decreasing frequency of PT according to all TEAE summary in Hylan G-F 20 treatment group

## Discussion

The results of this present study (C-SOUND trial) demonstrated that when compared with IA placebo, single 6 ml Hylan G-F 20 injection did not show superiority in providing symptomatic pain relief over a period of 26 weeks. However, treatment of a single IA administration of single 6 ml Hylan G-F 20 injection provided patients with pain relief in the targeted knee OA when compared to baseline. Both single 6 ml Hylan G-F 20 injection and placebo group were well tolerated in patients with primary knee OA.

The SOUND trial, which was previously conducted in the European centers was very similar to the current C-SOUND study with respect to the design, objectives, and number of enrolled participants. Both the SOUND and C-SOUND trials were double-blind, multi-center, placebo-controlled randomized controlled trials (RCTs) that aimed to compare the effect of a single 6 ml Hylan G-F 20 injection to that of 6 ml IA-placebo. Additionally, both trials had similar primary efficacy endpoints, with C-SOUND using WOMAC A1 and SOUND using WOMAC A to measure change from baseline in pain scores over 26 weeks. In terms of efficacy, the SOUND trial showed significant reductions in WOMAC A scores from baseline to 26 weeks in both the treatment (− 37.8%) and placebo arms (− 29.3%), with a significantly higher reduction in the treatment arm compared to the placebo (*p* = 0.047) [8]. Although both the SOUND and C-SOUND trials were similar in study design, and both showed that single 6 ml Hylan G-F 20 injection was efficacious in reducing pain scores from baseline to evaluation time of study endpoint, C-SOUND trial showed a more prominent placebo effect that overshadowed the main treatment effect causing the results not to show statistical significance for superiority of single 6 ml Hylan G-F 20 injection when compared to IA placebo.

The superiority of three 2-ml Hylan G-F 20 injection versus IA-placebo has also been demonstrated in three RCTs [10, 15] prior to the C-SOUND study. A multi-center, randomized, double-blind controlled clinical trial was conducted to compare the efficacy and safety of 3 doses of 2 ml Hylan G-F 20 weekly injection, versus IA-saline in 110 European patients of chronic idiopathic knee OA [10]. Results of this trial showed that 3 doses of 2 ml Hylan G-F 20 weekly injection significantly outperformed placebo for all outcomes measured using a VAS, including pain during weight-bearing movement and pain during the most painful knee movement. In another randomized double blind multi-center clinical trial in North America [15], one (3 weeks) or two courses (6 weeks) of three weekly IA injections of 3 doses of 2 ml Hylan G-F 20 weekly injection compared to control injections in 120 knee OA patients, demonstrated that patients in the 3 doses of 2 ml Hylan G-F 20 weekly injection group outperformed the placebo arm in terms of reaching the efficacy endpoints, including mean change in weight-bearing pain, night pain, improvement of knee movement, and overall assessment in both patient-assessed and evaluator-assessed outcome measures (VAS) at the 12-week endpoint. Hylan G-F 20’s superiority compared to various other comparator treatments such as NSAIDs [22, 23], conventional treatment [24, 25], intra-articular cortico-steroids [26], and other viscosupplementation therapies [27–29] has also been demonstrated in several other RCTs for the management of pain associated with knee OA. Data on the efficacy of Hylan G-F 20 from these RCTs are further supported by the results of various real-world evidence studies [25, 30–37], systematic reviews and meta-analyses [37–40]. The new evidence complemented and reinforced majority of evidence supporting the performances. Notably, the emergence of two recent studies on US claims databases of over 4.7 million patients (collectively) performed in parallel to the C-SOUND trial provided further insight on the performances of 3 doses of 2 ml Hylan G-F 20 weekly injection, indicating a positive effect in delaying the time to knee arthroplasty [34] and reducing patient need for opioids and IA-corticosteroids [41, 42], as well as adverse events associated with these treatments.

While the treatment effects from the baseline in the single 6 ml Hylan G-F 20 injection arm (WOMAC A1 scores: − 40.5%) in the C-SOUND trial were consistent with those observed in the SOUND trial (WOMAC A scores:-37.8%), the reduction in WOMAC A scores from baseline to 26 weeks in the placebo arm (change in WOMAC A1 scores: − 43.7%) of C-SOUND was substantially more pronounced compared to the SOUND trial (− 29.3%). The differences in patient characteristics and cultural environment may have allowed for patients living in China versus outside China to experience an overshadowing placebo effect which is higher than that observed in the European patients. Previous studies have shown that the administration of an IA placebo injection has yielded a statistically and clinically meaningful improvement in patients up to 6 months after the injection in patients with knee OA [10, 15]. Some studies have associated the prominent placebo effect, partly to the dilution of inflammatory mediators by the IA saline within the knee, providing relief of perceived pain and subjective stiffness [43]. Others, hypothesize this to stem from a complex interplay of sociopsychological factors, including the therapeutic ritual of receiving a perceived treatment, the interaction between patient and health care provider, the clinician’s confidence in the treatment, the patient’s personality effects, the method and frequency of substance administration, and the expectation for improvement by the patient [44–46]. The true biological and disease-modifying effect of placebo varies based on the disease and organ system but largely remains unknown [43]. Furthermore, meta-analysis conducted by Bannuru et al. and Altman et al. demonstrated that IA saline solution injections may not be without therapeutic effect but are active agents as their effect size of IA saline is comparable with oral NSAIDs. As a result, using IA saline solution as a control in a randomized control trial might diminish the effects of IA HA injections. Future clinical trials should also compare IA HA with sham injection and other nonsurgical treatments such as exercise, weight loss, and physical therapy [16, 47, 48]. Including an objective outcome measure, such as the 6-min walk test, which has been found to be an excellent predictor of functional performance after total-knee arthroplasty, may be able to capture treatment effects in future studies [49, 50].

In the C-SOUND trial, both single 6 ml Hylan G-F 20 injection and placebo were well tolerated in Chinese patients with primary knee OA. The number of treatment-related TEAEs in target knee was similar in the two treatment groups. The most commonly reported treatment-related TEAE in the target knee was joint swelling and arthralgia in the single 6 ml Hylan G-F 20 injection and in the placebo groups, respectively. This is in accordance with previous clinical trials [8, 10, 15].

There are several limitations which may hinder the validity of our results. The scales such as WOMAC A1, WOMAC A, PTGA and COGA should be well validated in the population living in China. Another limitation is that the current study was conducted in patients residing only within China and the results may not be applicable to patients outside China.

## Conclusions

While the relief of pain and safety in the single 6 ml Hylan G-F 20 injection group are consistent with previous results of 3 doses of 2 ml Hylan G-F 20 weekly injection studies, this study shows similar efficacy when comparing single 6 ml Hylan G-F 20 injection to IA placebo which may be due to a strong IA placebo effect. The findings of this clinical trial bring into question the relevance of this trial’s results to the published results of previously conducted trials where ethnicity may not have been considered as an important factor which may influence the results of a clinical trial investigating an active IA treatment in comparison with IA placebo in patients with knee OA. More studies are needed to investigate the effect of placebo in knee osteoarthritis in different part of the world and to evaluate impact of the social and cultural aspects when using IA placebo as a comparator in knee osteoarthritis clinical trials.

## Supplementary Information

**Additional file 1.** CONSORT 2010 checklist.

## Data Availability

Qualified researchers may request access to patient level data and related study documents including the clinical study report, study protocol with any amendments, blank case report form, statistical analysis plan, and dataset specifications. Patient level data will be anonymized, and study documents will be redacted to protect the privacy of trial participants. Dr. Yao Xie will be the contact person *(**yao.xie@sanofi.com**).* Further details on Sanofi’s data sharing criteria, eligible studies, and process for requesting access can be found at: *https://www.clinicalstudydatarequest.com**.*

## Abbreviations

ACR: American College of Rheumatology
ANCOVA: Analysis of covariance
COGA: Clinical observer global assessment
EQ-5D: European Quality of Life-5 Dimensions
GEE: Generalized estimating eq.
HA: Hyaluronic acid
IA: Intra-articular
IVRS: Interactive voice response system
LS: Least squares
mITT: Modified intent-to-treat
OA: Knee osteoarthritis
PTGA: Patient global assessment
PHQ-9: Patient Health Questionnaire-9
SD: Standard deviation
SE: Standard error
SOC: System-organ-class
TEAEs: Treatment-emergent adverse events
WOMAC: Western Ontario and McMaster Universities

## References

[1] Bijlsma JW, Berenbaum F, Lafeber FP (2011). Osteoarthritis: an update with relevance for clinical practice. Lancet..

[2] Bannuru RR, Vaysbrot EE, Sullivan MC, McAlindon TE (2014). Relative efficacy of hyaluronic acid in comparison with NSAIDs for knee osteoarthritis: a systematic review and meta-analysis. Semin Arthritis Rheum.

[3] D’Ambrosia RD (2005). Epidemiology of osteoarthritis. Orthopedic..

[4] Magnussen R, Mansour A, Carey J, Spindler K (2009). Meniscus status at anterior cruciate ligament reconstruction associated with radiographic signs of osteoarthritis at 5- to 10-year follow-up – a systematic review. J Knee Surg..

[5] Heidari B (2011). Knee osteoarthritis prevalence, risk factors, pathogenesis and features: part I. Caspian J Intern Med.

[6] Rillo O, Riera H, Acosta C, Liendo V, Bolaños J, Monterola L, et al. PANLAR consensus recommendations for the management in osteoarthritis of hand, hip, and knee. J Clin Rheumatol. 2016;22(7):345–54. 10.1097/RHU.0000000000000449. 10.1097/RHU.0000000000000449 27660931

[7] Cubukçu D, Ardiç F, Karabulut N, Topuz O (2005). Hylan G-F 20 efficacy on articular cartilage quality in patients with knee osteoarthritis: clinical and MRI assessment. Clin Rheumatol.

[8] Chevalier X, Jerosch J, Goupille P, van Dijk N, Luyten FP, Scott DL, et al. Single, intra-articular treatment with 6 ml hylan G-F 20 in patients with symptomatic primary osteoarthritis of the knee: a randomised, multicentre, double-blind, placebo controlled trial. Ann Rheum Dis. 2010;69(1):113–9. 10.1136/ard.2008.094623. 10.1136/ard.2008.094623 PMC2789938 19304567

[9] Scale D, Wobig M, Wolpert W (1994). Viscosupplementation of osteoarthritic knees with hylan: a treatment schedule study. Curr Ther Res.

[10] Wobig M, Dickhut A, Maier R, Vetter G (1998). Viscosupplementation with hylan G-F 20: a 26-week controlled trial of efficacy and safety in the osteoarthritic knee. Clin Ther.

[11] Zhang H, Zhang K, Zhang X, Zhu Z, Yan S, Sun T, et al. Comparison of two hyaluronic acid formulations for safety and efficacy (CHASE) study in knee osteoarthritis: a multicenter, randomized, double-blind, 26-week non-inferiority trial comparing Durolane to Artz. Arthritis Res Ther. 2015;17(1):51. 10.1186/s13075-015-0557-x. 10.1186/s13075-015-0557-x PMC4391669 25889322

[12] Altman RD, Akermark C, Beaulieu AD, Schnitzer T, Durolane International Study Group (2004). Efficacy and safety of a single intra-articular injection of non-animal stabilized hyaluronic acid (NASHA) in patients with osteoarthritis of the knee. Osteoarthr Cartil.

[13] Arden NK, Åkermark C, Andersson M, Todman MG, Altman RD (2014). A randomized saline-controlled trial of NASHA hyaluronic acid for knee osteoarthritis. Curr Med Res Opin.

[14] Altman RD, Manjoo A, Fierlinger A, Niazi F, Nicholls M (2015). The mechanism of action for hyaluronic acid treatment in the osteoarthritic knee: a systematic review. BMC Musculoskelet Disord.

[15] Moreland LW, Arnold JW, Saway A, Savory C, Sikes D (1993). Efficacy and safety of intra-articular hylan G-F 20 (Synvisc), a viscoelastic derivative of hyaluronan in patients with osteoarthritis of the knee. Arthritis Rheum.

[16] Conrozier T, Jerosch J, Beks P, Kemper F, Euller-Ziegler L, Bailleul F, et al. Prospective, multi-centre, randomised evaluation of the safety and efficacy of five dosing regimens of viscosupplementation with hylan G-F 20 in patients with symptomatic tibio-femoral osteoarthritis: a pilot study. Arch Orthop Trauma Surg. 2009;129(3):417–23. 10.1007/s00402-008-0601-2. 10.1007/s00402-008-0601-2 18365224

[17] Altman R, Asch E, Bloch D, Bole G, Borenstein D, Brandt K, et al. Development of criteria for the classification and reporting of osteoarthritis. Classification of osteoarthritis of the knee. Diagnostic and Therapeutic Criteria Committee of the American Rheumatism Association. Arthritis Rheum. 1986;29(8):1039–49. 10.1002/art.1780290816. 10.1002/art.1780290816 3741515

[18] Chinese Orthopedic Association (2007). Diagnosis and treatment of osteoarthritis guideline. Chin J Orthop.

[19] Levis B, Benedetti A, Thombs BD, DEPRESsion Screening Data (DEPRESSD) Collaboration (2019). Accuracy of Patient Health Questionnaire-9 (PHQ-9) for screening to detect major depression: individual participant data meta-analysis. BMJ.

[20] https://www.fda.gov/media/93884/download, March 2018 Procedural.

[21] Pham T, van der Heijde D, Altman RD, Anderson JJ, Bellamy N, Hochberg M, et al, Dougados M, OMERACT-OARSI Initiative: Osteoarthritis Research Society International set of responder criteria for osteoarthritis clinical trials revisited.. Osteoarthritis Cartilage. 2004;12(5):389-99. 10.1016/j.joca.2004.02.001. 10.1016/j.joca.2004.02.001 15094138

[22] Adams ME, Atkinson MH, Lussier AJ, Schulz JI, Siminovitch KA, Wade JP, et al. The role of viscosupplementation with hylan G-F 20 (Synvisc) in the treatment of osteoarthritis of the knee: a Canadian multicenter trial comparing hylan G-F 20 alone, hylan G-F 20 with non-steroidal anti-inflammatory drugs (NSAIDs) and NSAIDs alone. Osteoarthr Cartil. 1995;3(4):213–25. 10.1016/s1063-4584(05)80013-5. 10.1016/s1063-4584(05)80013-5 8689457

[23] Dickson DJ, Hosie G, English JR (2001). A double-blind, placebo-controlled comparison of hylan G-F 20 against diclofenac in knee osteoarthritis. J Drug Assess.

[24] Raynauld JP, Torrance GW, Band PA, Goldsmith CH, Tugwell P, Walker V, et al. A prospective, randomized, pragmatic, health outcomes trial evaluating the incorporation of hylan G-F 20 into the treatment paradigm for patients with knee osteoarthritis (part 1 of 2): clinical results. Osteoarthr Cartil. 2002;10(7):506–17. 10.1053/joca.2002.0798. 10.1053/joca.2002.0798 12127830

[25] Hermans J, Bierma-Zeinstra SMA, Bos PK, Niesten DD, Verhaar JAN, Reijman M (2019). The effectiveness of high molecular weight hyaluronic acid for knee osteoarthritis in patients in the working age: a randomised controlled trial. BMC Musculoskelet Disord.

[26] Caborn D, Rush J, Lanzer W, Parenti D, Murray C, Synvisc 901 Study Group (2004). A randomized, single-blind comparison of the efficacy and tolerability of hylan G-F 20 and triamcinolone hexacetonide in patients with osteoarthritis of the knee. J Rheumatol.

[27] Al-Omran A, Azam Q (2014). Efficacy of viscosupplementation in knee osteoarthritis: a clinical trial of three agents. Bahrain Med Bull.

[28] Raman R, Dutta A, Day N, Sharma HK, Shaw CJ, Johnson GV (2008). Efficacy of Hylan G-F 20 and sodium hyaluronate in the treatment of osteoarthritis of the knee -- a prospective randomized clinical trial. Knee..

[29] Wobig M, Bach G, Beks P, Dickhut A, Runzheimer J, Schwieger G, et al. The role of elastoviscosity in the efficacy of viscosupplementation for osteoarthritis of the knee: a comparison of hylan G-F 20 and a lower-molecular-weight hyaluronan. Clin Ther. 1999;21(9):1549–62. 10.1016/S0149-2918(00)80010-7. 10.1016/s0149-2918(00)80010-7 10509850

[30] Kemper F, Gebhardt U, Meng T, Murray C (2005). Tolerability and short-term effectiveness of hylan G-F 20 in 4253 patients with osteoarthritis of the knee in clinical practice. Curr Med Res Opin.

[31] Petrella RJ, Wakeford C (2015). Pain relief and improved physical function in knee osteoarthritis patients receiving ongoing hylan G-F 20, a high-molecular-weight hyaluronan, versus other treatment options: data from a large real-world longitudinal cohort in Canada. Drug Des Devel Ther.

[32] Yan CH, Chan WL, Yuen WH, Yung PS, Ip KY, Fan JC (2015). Efficacy and safety of hylan G-F 20 injection in treatment of knee osteoarthritis in Chinese patients: results of a prospective, multicentre, longitudinal study. Hong Kong Med J.

[33] Waddell D, Joseph B (2016). Delayed total knee replacement with Hylan G-F 20. J Knee Surg.

[34] Ong KL, Runa M, Lau E, Altman R (2019). Is intra-articular injection of synvisc associated with a delay to knee arthroplasty in patients with knee osteoarthritis?. Cartilage..

[35] Wobig M, Beks P, Dickhut A, Maier R, Vetter G (1999). Open-label multicenter trial of the safety and efficacy of viscosupplementation with Hylan G-F 20 (Synvisc) in primary osteoarthritis of the knee. JCR J Clin Rheumatol.

[36] Pal S, Thuppal S, Reddy KJ, Avasthi S, Aggarwal A, Bansal H, et al. Long-term (1-year) safety and efficacy of a single 6-mL injection of Hylan G-F 20 in Indian patients with symptomatic knee osteoarthritis. Open Rheumatol J. 2014;8(1):54–68. 10.2174/1874312901408010054. 10.2174/1874312901408010054 PMC4196249 25328555

[37] Chevalier X, Sheehan B, Whittington C, Ho C, Ngai W, Campos G (2019). Efficacy and Safety of Hylan GF 20 versus Intra-Articular Corticosteroids in Patients with Knee Osteoarthritis: A Systematic Literature Review, Meta-Analysis, and Network Meta-analysis.

[38] Bellamy N, Campbell J, Robinson V, Gee T, Bourne R, Wells G (2005). Viscosupplementation for the treatment of osteoarthritis of the knee. Cochrane Database Syst Rev.

[39] Brander VA, Stadler TS (2009). Functional improvement with hylan G-F 20 in patients with knee osteoarthritis. Phys Sportsmed.

[40] Zhao H, Liu H, Liang X, Li Y, Wang J, Liu C (2016). Hylan G-F 20 versus low molecular weight hyaluronic acids for knee osteoarthritis: a meta-analysis. BioDrugs..

[41] Webner D, Langworthy M, Ngai W, Hao L, Hummer C (2019). Retrospective analysis of opioid prescription surrounding treatment with Hylan G-F 20 in patients with osteoarthritis of the knee. Paper presented at: PAIN Week 2019.

[42] Webner D, Langworthy M, Ngai W, Hao LCH. Retrospective analysis of intra-articular corticosteroid injections surrounding treatment with Hylan G-F 20 in patients with osteoarthritis of the knee. Paper presented at: PAIN Week 2019; 2019.

[43] Saltzman BM, Leroux T, Meyer MA, Basques BA, Chahal J, Bach BR Jr, et al. The therapeutic effect of intra-articular normal saline injections for knee osteoarthritis: a meta-analysis of evidence level 1 studies. Am J Sports Med. 2017;45(11):2647–53. 10.1177/0363546516680607. 10.1177/0363546516680607 28027657

[44] Huang SM, Temple R (2008). Is this the drug or dose for you? Impact and consideration of ethnic factors in global drug development, regulatory review, and clinical practice. Clin Pharmacol Ther.

[45] Abhishek A, Doherty M (2013). Mechanisms of the placebo response in pain in osteoarthritis. Osteoarthr Cartil.

[46] De Campos GC (2015). Placebo effect in osteoarthritis: why not use it to our advantage?. World J Orthop.

[47] Bannuru RR, McAlindon TE, Sullivan MC, Wong JB, Kent DM, Schmid CH (2015). Effectiveness and implications of alternative placebo treatments: a systematic review and network meta-analysis of osteoarthritis trials. Ann Intern Med.

[48] Altman RD, Devji T, Bhandari M, Fierlinger A, Niazi F, Christensen R (2016). Clinical benefit of intra-articular saline as a comparator in clinical trials of knee osteoarthritis treatments: a systematic review and meta-analysis of randomized trials. Semin Arthritis Rheum.

[49] Rikli RE, Jones CJ (1998). The reliability and validity of a 6-minute walk test as a measure of physical endurance in older adults. J Aging Phys Act.

[50] Ko V, Naylor JM, Harris IA, Crosbie J, Yeo AE. The six-minute walk test is an excellent predictor of functional ambulation after total knee arthroplasty. BMC Musculoskelet Disord. 2013;14(1):145. 10.1186/1471-2474-14-145 PMC3644243 23617377

